# Methyl 2-(5-chloro-3-methyl­sulfinyl-1-benzofuran-2-yl)acetate

**DOI:** 10.1107/S1600536808033503

**Published:** 2008-10-18

**Authors:** Hong Dae Choi, Pil Ja Seo, Byeng Wha Son, Uk Lee

**Affiliations:** a Department of Chemistry, Dongeui University, San 24 Kaya-dong, Busanjin-gu, Busan 614-714, Republic of Korea; b Department of Chemistry, Pukyong National University, 599-1 Daeyeon 3-dong, Nam-gu, Busan 608-737, Republic of Korea

## Abstract

The title compound, C_12_H_11_ClO_4_S, was prepared by the oxidation of methyl 2-(5-chloro-3-methyl­sulfanyl-1-benzofuran-2-yl)acetate with 3-chloro­peroxy­benzoic acid. The O atom and the methyl group of the methyl­sulfinyl substituent lie on opposite sides of the plane of the benzofuran fragment. The crystal structure is stabilized by aromatic π–π inter­actions between the benzene rings of neighbouring mol­ecules [centroid-to-centroid distance = 3.809 (2) Å], and by C—H⋯π inter­actions between a methyl H atom and the furan ring of an adjacent mol­ecule. In addition, the crystal structure exhibits inter­molecular C—H⋯O hydrogen bonds.

## Related literature

For details of the pharmacological activities of benzofuran compounds, see: Ward (1999 ▶). For the crystal structures of similar 2-(3-methyl­sulfinyl-1-benzofuran-2-yl)acetic acid derivatives, see: Choi *et al.* (2007 ▶, 2008 ▶).
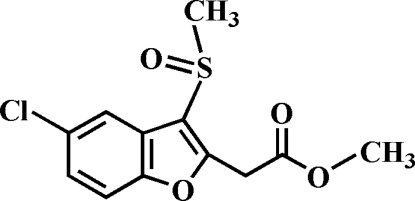


## Experimental

### 

#### Crystal data

C_12_H_11_ClO_4_S
*M* *_r_* = 286.72
Triclinic, 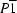

*a* = 7.8910 (5) Å
*b* = 8.9416 (6) Å
*c* = 10.4048 (7) Å
α = 73.774 (1)°
β = 78.743 (1)°
γ = 68.559 (1)°
*V* = 652.55 (7) Å^3^
*Z* = 2
Mo *K*α radiation
μ = 0.46 mm^−1^
*T* = 298 (2) K
0.40 × 0.40 × 0.20 mm

#### Data collection

Bruker SMART CCD diffractometer
Absorption correction: multi-scan (*SADABS*; Sheldrick, 1999 ▶) *T*_min_ = 0.827, *T*_max_ = 0.907
3753 measured reflections
2525 independent reflections
2123 reflections with *I* > 2σ(*I*)
*R*_int_ = 0.012

#### Refinement

*R*[*F* ^2^ > 2σ(*F* ^2^)] = 0.035
*wR*(*F* ^2^) = 0.101
*S* = 1.06
2525 reflections
169 parameters
H atoms treated by a mixture of independent and constrained refinement
Δρ_max_ = 0.23 e Å^−3^
Δρ_min_ = −0.25 e Å^−3^

### 

Data collection: *SMART* (Bruker, 2001 ▶); cell refinement: *SAINT* (Bruker, 2001 ▶); data reduction: *SAINT*; program(s) used to solve structure: *SHELXS97* (Sheldrick, 2008 ▶); program(s) used to refine structure: *SHELXL97* (Sheldrick, 2008 ▶); molecular graphics: *ORTEP-3* (Farrugia, 1997 ▶) and *DIAMOND* (Brandenburg, 1998 ▶); software used to prepare material for publication: *SHELXL97*.

## Supplementary Material

Crystal structure: contains datablocks global, I. DOI: 10.1107/S1600536808033503/zl2153sup1.cif

Structure factors: contains datablocks I. DOI: 10.1107/S1600536808033503/zl2153Isup2.hkl

Additional supplementary materials:  crystallographic information; 3D view; checkCIF report

## Figures and Tables

**Table 1:** Hydrogen-bond geometry (Å, °) *Cg*1 is the centroid of the C1/C2/C7/O1/C8 furan ring.

*D*—H⋯*A*	*D*—H	H⋯*A*	*D*⋯*A*	*D*—H⋯*A*
C11—H11*C*⋯*Cg*1^i^	0.96	2.92	3.858 (2)	165
C3—H3⋯O4^ii^	0.94 (2)	2.41 (2)	3.320 (3)	162.7 (17)
C9—H9*B*⋯O1^iii^	0.97	2.59	3.550 (2)	172
C9—H9*A*⋯O4^iv^	0.97	2.23	3.183 (3)	169

Symmetry codes: (i) 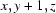; (ii) 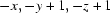; (iii) 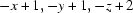; (iv) 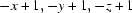.

**Fig. 1.**
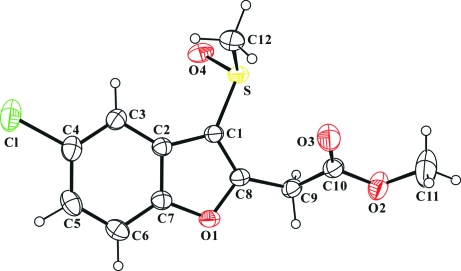
The molecular structure of the title compound, showing displacement ellipsoids drawn at the 30% probability level.

**Fig. 2.**
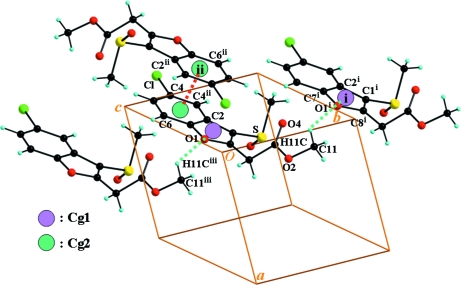
π–π and C—H···π interactions (dotted lines) in the title compound. Cg denotes the ring centroid. [Symmetry code: (i) x, y + 1, z; (ii) -x, -y + 1, -z + 2; (iii) x, y - 1, z.]

## supplementary crystallographic information

### Comment

Benzofuran derivatives occur widely in natural products and show a variety of interesting pharmacological activities such as antimicrobial, fungicidal, insecticidal and antioxidant properties (Ward, 1999). As a part of our ongoing studies on the synthesis and structure of 2-(3-methylsulfinyl-1-benzofuran-2-yl)acetic acid analogues, the crystal structure of ethyl 2-(5-chloro-3-methylsulfinyl-1-benzofuran-2-yl)acetate (Choi *et al.*, 2007) and methyl 2-(5-methyl-3-methylsulfinyl-1-benzofuran-2-yl)acetate (Choi *et al.*, 2008) have been described in the literature. Here we report the crystal structure of the title compound, methyl 2-(5-chloro-3-methylsulfinyl-1-benzofuran-2-yl)acetate (Fig. 1).

The benzofuran unit is essentially planar, with a mean deviation of 0.011 (2) Å from the least-squares plane defined by the nine constituent atoms. The molecular packing (Fig. 2) is stabilized by aromatic π–π stacking interactions between the benzene rings of adjacent molecules. The *Cg*2···*Cg*2^ii^ distance is 3.809 (2) Å (*Cg*2 is the centroid of the C2–C7 benzene ring; symmetry code as in Fig. 2). The molecular packing is further stabilized by C—H···π interactions between a methyl H atom and the furan ring of the benzofuran uint, with a C11—H11C···*Cg*1^i^ separation of 2.92 Å (Fig. 2 and Table 1; *Cg*1 is the centroid of the C1/C2/C7/O1/C8 furan ring). Additionally, intermolecular C—H···O hydrogen bonds in the structure were observed (Table 1).

### Experimental

77% 3-Chloroperoxybenzoic acid (292 mg, 1.3 mmol) was added in small portions to a stirred solution of methyl 2-(5-chloro-3-methylsulfanyl-1-benzofuran-2-yl)acetate (325 mg, 1.2 mmol) in dichloromethane (40 ml) at 273 K. After being stirred for 3 h at room temperature, the mixture was washed with saturated sodium bicarbonate solution and the organic layer was separated, dried over magnesium sulfate, filtered and concentrated in vacuum. The residue was purified by column chromatography (ethyl acetate) to afford the title compound as a colorless solid [yield 77%, m.p. 415–416 K; *R*_f_ = 0.72 (ethyl acetate)]. Single crystals suitable for X-ray diffraction were prepared by evaporation of a solution of the title compound in benzene at room temperature. Spectroscopic analysis: ^1^H NMR (CDCl_3_, 400 MHz) δ 3.07 (s, 3H), 3.75 (s, 3H), 4.05 (s, 2H), 7.34 (dd, J = 8.76 Hz and J = 1.80 Hz, 1H), 7.44 (d, J = 8.80 Hz, 1H), 7.90 (d, J = 1.80 Hz, 1H); EI–MS 288 [*M*+2], 286[*M*^+^].

### Refinement

All H atoms were geometrically positioned and refined using a riding model, with C—H = 0.93 Å (aromatic), 0.97 Å (methylene), and 0.96 Å (methyl) H atoms, respectively, and with *U*_iso_(H) = 1.2Ueq(C) (aromatic & methylene), and 1.5Ueq(C) (methyl) H atoms.

### Crystal data

**Table:**

C_12_H_11_ClO_4_S	*Z* = 2
*M**_r_* = 286.72	*F*(000) = 296
Triclinic, *P*1	*D*_x_ = 1.459 Mg m^−^^3^
Hall symbol: -P_1	Melting point = 415–416 K
*a* = 7.8910 (5) Å	Mo *K*α radiation, λ = 0.71073 Å
*b* = 8.9416 (6) Å	Cell parameters from 2313 reflections
*c* = 10.4048 (7) Å	θ = 2.8–28.2°
α = 73.774 (1)°	µ = 0.46 mm^−^^1^
β = 78.743 (1)°	*T* = 298 K
γ = 68.559 (1)°	Block, colourless
*V* = 652.55 (7) Å^3^	0.40 × 0.40 × 0.20 mm

### Data collection

**Table:**

Bruker SMART CCD diffractometer	2525 independent reflections
Radiation source: fine-focus sealed tube	2123 reflections with *I* > 2σ(*I*)
graphite	*R*_int_ = 0.012
Detector resolution: 10.0 pixels mm^-1^	θ_max_ = 26.0°, θ_min_ = 2.5°
φ and ω scans	*h* = −9→9
Absorption correction: multi-scan (SADABS; Sheldrick, 1999)	*k* = −11→7
*T*_min_ = 0.827, *T*_max_ = 0.907	*l* = −12→12
3753 measured reflections	

### Refinement

**Table:**

Refinement on *F*^2^	Primary atom site location: structure-invariant direct methods
Least-squares matrix: full	Secondary atom site location: difference Fourier map
*R*[*F*^2^ > 2σ(*F*^2^)] = 0.035	Hydrogen site location: inferred from neighbouring sites
*wR*(*F*^2^) = 0.101	H atoms treated by a mixture of independent and constrained refinement
*S* = 1.06	*w* = 1/[σ^2^(*F*_o_^2^) + (0.0497*P*)^2^ + 0.2053*P*] where *P* = (*F*_o_^2^ + 2*F*_c_^2^)/3
2525 reflections	(Δ/σ)_max_ < 0.001
169 parameters	Δρ_max_ = 0.23 e Å^−^^3^
0 restraints	Δρ_min_ = −0.25 e Å^−^^3^

### Special details

**Table:**

Geometry. All e.s.d.'s (except the e.s.d. in the dihedral angle between two l.s. planes) are estimated using the full covariance matrix. The cell e.s.d.'s are taken into account individually in the estimation of e.s.d.'s in distances, angles and torsion angles; correlations between e.s.d.'s in cell parameters are only used when they are defined by crystal symmetry. An approximate (isotropic) treatment of cell e.s.d.'s is used for estimating e.s.d.'s involving l.s. planes.
Refinement. Refinement of *F*^2^ against ALL reflections. The weighted *R*-factor *wR* and goodness of fit *S* are based on *F*^2^, conventional *R*-factors *R* are based on *F*, with *F* set to zero for negative *F*^2^. The threshold expression of *F*^2^ > σ(*F*^2^) is used only for calculating *R*-factors(gt) *etc*. and is not relevant to the choice of reflections for refinement. *R*-factors based on *F*^2^ are statistically about twice as large as those based on *F*, and *R*- factors based on ALL data will be even larger.

### Fractional atomic coordinates and isotropic or equivalent isotropic displacement parameters (Å^2^)

**Table:**

	*x*	*y*	*z*	*U*_iso_*/*U*_eq_	
Cl	−0.22541 (8)	0.20560 (7)	0.87139 (8)	0.0834 (2)	
S	0.23616 (7)	0.63233 (7)	0.54121 (5)	0.05176 (17)	
O1	0.33659 (17)	0.45642 (16)	0.92043 (12)	0.0468 (3)	
O2	0.5278 (2)	0.89402 (19)	0.72451 (19)	0.0728 (5)	
O3	0.2521 (2)	0.9006 (2)	0.69823 (19)	0.0748 (5)	
O4	0.2462 (2)	0.4995 (2)	0.47609 (15)	0.0688 (4)	
C1	0.2400 (2)	0.5423 (2)	0.71481 (17)	0.0429 (4)	
C2	0.1418 (2)	0.4347 (2)	0.79676 (17)	0.0424 (4)	
C3	0.0067 (3)	0.3789 (2)	0.7780 (2)	0.0489 (4)	
H3	−0.040 (3)	0.409 (3)	0.694 (2)	0.053 (6)*	
C4	−0.0519 (3)	0.2747 (2)	0.8878 (2)	0.0555 (5)	
C5	0.0180 (3)	0.2229 (3)	1.0116 (2)	0.0596 (5)	
H5	−0.0254	0.1511	1.0818	0.072*	
C6	0.1517 (3)	0.2778 (2)	1.0307 (2)	0.0556 (5)	
H6	0.2007	0.2447	1.1124	0.067*	
C7	0.2087 (2)	0.3845 (2)	0.92181 (19)	0.0450 (4)	
C8	0.3537 (2)	0.5507 (2)	0.79220 (18)	0.0433 (4)	
C9	0.4870 (3)	0.6396 (2)	0.7667 (2)	0.0494 (4)	
H9A	0.5801	0.6024	0.6959	0.059*	
H9B	0.5470	0.6105	0.8475	0.059*	
C10	0.4045 (3)	0.8245 (3)	0.72678 (19)	0.0489 (4)	
C11	0.4682 (5)	1.0733 (3)	0.6855 (4)	0.1092 (12)	
H11A	0.4158	1.1104	0.6016	0.164*	
H11B	0.5713	1.1098	0.6754	0.164*	
H11C	0.3782	1.1180	0.7537	0.164*	
C12	0.0024 (3)	0.7661 (3)	0.5437 (2)	0.0661 (6)	
H12A	−0.0223	0.8297	0.4543	0.099*	
H12B	−0.0183	0.8390	0.6018	0.099*	
H12C	−0.0772	0.7011	0.5764	0.099*	

### Atomic displacement parameters (Å^2^)

**Table:**

	*U*^11^	*U*^22^	*U*^33^	*U*^12^	*U*^13^	*U*^23^
Cl	0.0594 (4)	0.0550 (3)	0.1360 (6)	−0.0274 (3)	−0.0214 (4)	−0.0029 (4)
S	0.0541 (3)	0.0652 (3)	0.0390 (3)	−0.0251 (2)	−0.0121 (2)	−0.0044 (2)
O1	0.0496 (7)	0.0499 (7)	0.0406 (7)	−0.0133 (6)	−0.0141 (5)	−0.0075 (5)
O2	0.0579 (9)	0.0575 (9)	0.1130 (14)	−0.0244 (7)	−0.0186 (9)	−0.0208 (9)
O3	0.0557 (9)	0.0564 (9)	0.1072 (13)	−0.0179 (7)	−0.0286 (9)	0.0023 (9)
O4	0.0751 (10)	0.0872 (11)	0.0505 (8)	−0.0219 (9)	−0.0140 (7)	−0.0269 (8)
C1	0.0460 (9)	0.0439 (10)	0.0394 (9)	−0.0139 (8)	−0.0101 (7)	−0.0081 (7)
C2	0.0436 (9)	0.0383 (9)	0.0431 (9)	−0.0087 (7)	−0.0086 (7)	−0.0096 (7)
C3	0.0465 (10)	0.0432 (10)	0.0584 (12)	−0.0125 (8)	−0.0119 (9)	−0.0123 (9)
C4	0.0449 (10)	0.0376 (10)	0.0800 (14)	−0.0101 (8)	−0.0095 (10)	−0.0100 (9)
C5	0.0537 (12)	0.0419 (10)	0.0671 (13)	−0.0109 (9)	0.0000 (10)	0.0018 (9)
C6	0.0564 (12)	0.0489 (11)	0.0489 (11)	−0.0091 (9)	−0.0087 (9)	−0.0009 (9)
C7	0.0437 (9)	0.0416 (10)	0.0455 (10)	−0.0078 (8)	−0.0086 (7)	−0.0093 (8)
C8	0.0428 (9)	0.0448 (10)	0.0417 (9)	−0.0107 (8)	−0.0107 (7)	−0.0093 (7)
C9	0.0449 (10)	0.0534 (11)	0.0534 (11)	−0.0163 (8)	−0.0143 (8)	−0.0115 (9)
C10	0.0472 (10)	0.0557 (11)	0.0468 (10)	−0.0193 (9)	−0.0064 (8)	−0.0124 (8)
C11	0.095 (2)	0.0580 (16)	0.186 (4)	−0.0338 (16)	−0.030 (2)	−0.0244 (19)
C12	0.0659 (13)	0.0603 (13)	0.0673 (14)	−0.0152 (11)	−0.0275 (11)	−0.0009 (11)

### Geometric parameters (Å, °)

**Table:**

Cl—C4	1.748 (2)	C4—C5	1.391 (3)
S—O4	1.4950 (17)	C5—C6	1.381 (3)
S—C1	1.7613 (18)	C5—H5	0.9300
S—C12	1.794 (2)	C6—C7	1.380 (3)
O1—C8	1.376 (2)	C6—H6	0.9300
O1—C7	1.377 (2)	C8—C9	1.482 (3)
O2—C10	1.327 (2)	C9—C10	1.504 (3)
O2—C11	1.454 (3)	C9—H9A	0.9700
O3—C10	1.194 (2)	C9—H9B	0.9700
C1—C8	1.350 (2)	C11—H11A	0.9600
C1—C2	1.443 (3)	C11—H11B	0.9600
C2—C7	1.394 (3)	C11—H11C	0.9600
C2—C3	1.396 (3)	C12—H12A	0.9600
C3—C4	1.378 (3)	C12—H12B	0.9600
C3—H3	0.94 (2)	C12—H12C	0.9600
			
O4—S—C1	105.98 (9)	C6—C7—C2	123.72 (18)
O4—S—C12	106.01 (11)	C1—C8—O1	110.86 (16)
C1—S—C12	98.50 (10)	C1—C8—C9	133.29 (17)
C8—O1—C7	106.35 (13)	O1—C8—C9	115.85 (15)
C10—O2—C11	116.4 (2)	C8—C9—C10	114.36 (15)
C8—C1—C2	107.57 (15)	C8—C9—H9A	108.7
C8—C1—S	124.18 (15)	C10—C9—H9A	108.7
C2—C1—S	127.95 (14)	C8—C9—H9B	108.7
C7—C2—C3	119.64 (17)	C10—C9—H9B	108.7
C7—C2—C1	104.63 (16)	H9A—C9—H9B	107.6
C3—C2—C1	135.72 (17)	O3—C10—O2	123.6 (2)
C4—C3—C2	116.31 (19)	O3—C10—C9	126.28 (19)
C4—C3—H3	121.9 (13)	O2—C10—C9	110.06 (16)
C2—C3—H3	121.8 (13)	O2—C11—H11A	109.5
C3—C4—C5	123.7 (2)	O2—C11—H11B	109.5
C3—C4—Cl	118.25 (17)	H11A—C11—H11B	109.5
C5—C4—Cl	118.09 (16)	O2—C11—H11C	109.5
C6—C5—C4	120.25 (19)	H11A—C11—H11C	109.5
C6—C5—H5	119.9	H11B—C11—H11C	109.5
C4—C5—H5	119.9	S—C12—H12A	109.5
C7—C6—C5	116.40 (19)	S—C12—H12B	109.5
C7—C6—H6	121.8	H12A—C12—H12B	109.5
C5—C6—H6	121.8	S—C12—H12C	109.5
O1—C7—C6	125.70 (17)	H12A—C12—H12C	109.5
O1—C7—C2	110.58 (15)	H12B—C12—H12C	109.5
			
O4—S—C1—C8	129.68 (17)	C5—C6—C7—C2	−1.4 (3)
C12—S—C1—C8	−120.88 (18)	C3—C2—C7—O1	−178.32 (15)
O4—S—C1—C2	−43.19 (19)	C1—C2—C7—O1	1.0 (2)
C12—S—C1—C2	66.26 (19)	C3—C2—C7—C6	1.4 (3)
C8—C1—C2—C7	−0.4 (2)	C1—C2—C7—C6	−179.31 (18)
S—C1—C2—C7	173.45 (14)	C2—C1—C8—O1	−0.4 (2)
C8—C1—C2—C3	178.8 (2)	S—C1—C8—O1	−174.49 (13)
S—C1—C2—C3	−7.4 (3)	C2—C1—C8—C9	−179.77 (19)
C7—C2—C3—C4	−0.2 (3)	S—C1—C8—C9	6.1 (3)
C1—C2—C3—C4	−179.2 (2)	C7—O1—C8—C1	1.0 (2)
C2—C3—C4—C5	−1.0 (3)	C7—O1—C8—C9	−179.52 (15)
C2—C3—C4—Cl	178.26 (14)	C1—C8—C9—C10	60.7 (3)
C3—C4—C5—C6	1.0 (3)	O1—C8—C9—C10	−118.61 (18)
Cl—C4—C5—C6	−178.22 (16)	C11—O2—C10—O3	0.5 (4)
C4—C5—C6—C7	0.2 (3)	C11—O2—C10—C9	179.1 (2)
C8—O1—C7—C6	179.08 (18)	C8—C9—C10—O3	−10.1 (3)
C8—O1—C7—C2	−1.22 (19)	C8—C9—C10—O2	171.31 (17)
C5—C6—C7—O1	178.32 (18)		

### Hydrogen-bond geometry (Å, °)

**Table:**

*D*—H···*A*	*D*—H	H···*A*	*D*···*A*	*D*—H···*A*
C11—H11C···Cg1^i^	0.96	2.92	3.858 (2)	165
C3—H3···O4^ii^	0.94 (2)	2.41 (2)	3.320 (3)	162.7 (17)
C9—H9B···O1^iii^	0.97	2.59	3.550 (2)	172
C9—H9A···O4^iv^	0.97	2.23	3.183 (3)	169

Symmetry codes: (i) *x*, *y*+1, *z*; (ii) −*x*, −*y*+1, −*z*+1; (iii) −*x*+1, −*y*+1, −*z*+2; (iv) −*x*+1, −*y*+1, −*z*+1.
